# 2411: A pilot study: Using computational fluid dynamics to model physiologic airflow through an ovine tissue engineered tracheal graft

**DOI:** 10.1017/cts.2017.231

**Published:** 2018-05-10

**Authors:** Nakesha King, Victoria Pepper, Cameron Best, Ekene Onwuka, Chengyu Li, Eric Heuer, Jed Johnson, Kai Zhao, Christopher K. Breuer, Tendy Chiang

**Affiliations:** The Research Institute at Nationwide Children’s Hospital, Columbus, OH, USA

## Abstract

OBJECTIVES/SPECIFIC AIMS: Tissue engineered tracheal grafts (TETG) could provide a life-saving cure for children with long segment airway defects. Computational fluid dynamics (CFD) is a novel and promising technique used to evaluate TETG performance. This pilot study examines the correlation of objective CFD simulations with subjective respiratory symptoms in a TETG large animal model. METHODS/STUDY POPULATION: Three-dimensional geometries of 1 TETG implanted sheep trachea were reconstructed from serial fluoroscopic images, allowing analysis with CFD simulations. Peak flow velocity (PFV) and peak wall shear stress (PWSS) across the graft as well as changes secondary to stenting were determined. CFD metrics were compared with respiratory symptoms seen on exam. RESULTS/ANTICIPATED RESULTS: Two weeks after implantation, the animal developed respiratory distress, which correlated with PFV and PWSS elevations. Although the intraluminal graft appearance changed minimally after dilation, PFV and PWSS decreased across the graft (4.5–0.8 m/s and 0.9–0.1 Pa, respectively). Long-term TETG stenting with dilation returned PFV and PWSS to baseline (0.8–0.3 m/s and 0.1–0.01 Pa, respectively), which correlated with immediate symptom resolution. DISCUSSION/SIGNIFICANCE OF IMPACT: CFD is a noninvasive modality, which allows the evaluation of airflow metrics of symptomatic TETG recipients. This diagnostic tool will permit planned interventions and graft design optimization.

